# Bioactive Compounds of Plant‐Based Food: Extraction, Isolation, Identification, Characteristics, and Emerging Applications

**DOI:** 10.1002/fsn3.70351

**Published:** 2025-06-11

**Authors:** Tarek Gamal Abedelmaksoud, Mohamed Ibrahim Younis, Ammar B. Altemimi, Rawaa H. Tlay, Nora Ali Hassan

**Affiliations:** ^1^ Food Science Department, Faculty of Agriculture Cairo University Giza Egypt; ^2^ Department of Food Science College of Agriculture, University of Basrah Basrah Iraq; ^3^ Department of Food Science College of Agriculture, Damascus University Damascus Syria

**Keywords:** bioactive compounds, characteristics, emerging applications, extraction, identification, isolation

## Abstract

This review delves into the diverse array of bioactive compounds present in plant‐based foods, emphasizing their extraction, isolation, identification, characteristics, and emerging applications. Plant‐based foods are abundant in bioactive compounds such as polyphenols, flavonoids, alkaloids, terpenoids, and carotenoids, which have gained substantial attention for their potential health benefits and functional properties. The extraction of these compounds employs both conventional methods, like solvent extraction, and modern techniques, including supercritical fluid extraction, ultrasound‐assisted extraction, and microwave‐assisted extraction, all designed to efficiently recover bioactive compounds while maintaining their structural integrity and bioactivity. Isolation processes, such as chromatography (HPLC, GC), centrifugation, and filtration, are employed to separate and purify these compounds from complex plant matrices. Advanced analytical methods, including mass spectrometry (MS), nuclear magnetic resonance (NMR), and infrared spectroscopy (IR), are used for the identification and structural elucidation of these bioactive compounds, providing detailed insights into their chemical makeup and properties. The characteristics of these compounds, such as antioxidant, anti‐inflammatory, antimicrobial, and anticancer activities, have been extensively studied for their therapeutic potential. Additionally, the growing applications of bioactive compounds in functional foods, nutraceuticals, pharmaceuticals, and cosmeceuticals underscore their expanding significance across various industries. This review offers a thorough exploration of bioactive compounds in plant‐based foods, covering their extraction and isolation methods, identification, properties, and emerging uses, underscoring their vast potential to enhance human health and well‐being.

## Graphical abstract

This review highlights the abundance of bioactive compounds in plant‐based foods, such as polyphenols, flavonoids, and carotenoids, emphasizing their health benefits and functional properties. It discusses advanced extraction, isolation, and analytical methods, along with the therapeutic activities of these compounds, and explores their applications in functional foods, nutraceuticals, and pharmaceuticals. The findings underscore the transformative potential of bioactive compounds in promoting health and advancing various industries.

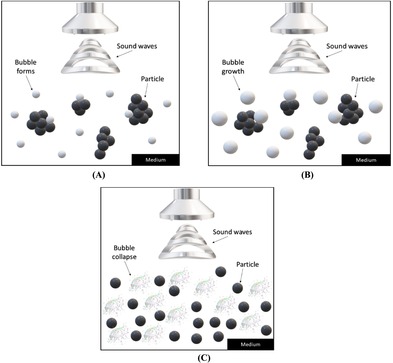

## 1 Introduction

Plant‐derived food includes a plethora of bioactive compounds, which are inherent substances with potential health benefits. The aforementioned substances, including polyphenols, carotenoids, flavonoids, and alkaloids, demonstrate a wide range of characteristics, encompassing antioxidant activities (Thuphairo et al. 2019), anti‐inflammatory (Villarreal‐Soto et al. 2019), anticancer (Wu et al. 2019), and antimicrobial effects (Gosset‐Erard et al. 2021). The growing recognition of the health advantages linked to bioactive compounds has led to a notable upswing in research focusing on the extraction, isolation, identification, and characterization of these compounds derived from plant‐based dietary sources. Furthermore, other businesses, such as the food, pharmaceutical, and cosmetics sectors are displaying significant enthusiasm in investigating the possible uses of these bioactive substances (Shang et al. 2019). In recent years, significant advancements have been observed in the sector, principally attributed to the emergence of advanced methodologies and technologies employed in the extraction, isolation, and characterization of bioactive substances (Essien et al. 2020). This advancement has resulted in a more profound comprehension of the chemical compositions, properties, and prospective physiological advantages of these substances, hence facilitating their utilization across diverse sectors. Nevertheless, despite the notable progress, there are still persistent obstacles associated with the extraction, isolation, and identification procedures of bioactive compounds obtained from plant‐based dietary sources (Mahato et al. 2019). This review aims to present a comprehensive overview of the extraction, isolation, identification, characteristics, and emerging applications of bioactive compounds obtained from plant‐based foods. This article will explore several methodologies and technologies utilized in the extraction and isolation of these substances while analyzing their chemical structures, characteristics, and possible implications for human health. Furthermore, this study will investigate the rising applications of bioactive compounds in diverse sectors, with a particular focus on the obstacles that need to be addressed in order to fully exploit the capabilities of these substances.

## 2 Techniques for the Extraction of Bioactive Compounds from Plant‐Based Foods

### 2.1 Solvent Extraction

The process of extracting bioactive substances from plant‐based food is a complex procedure that requires the use of several solvents to efficiently dissolve and extract these compounds. Solvent extraction is a highly prevalent technology in several industries, owing to its inherent advantages in terms of simplicity, efficiency, and cost‐effectiveness. The choice of a suitable solvent for the extraction of bioactive compounds is contingent upon several aspects, including the kind and features of the solvent, its solubility in the selected solvent, and the possible environmental effect and toxicity of the solvent.

Water, ethanol, methanol, acetone, and hexane are among the commonly utilized solvents for bioactive compound extraction. Water, being a polar solvent, is commonly employed for the extraction of polar substances, including phenolic acids and flavonoids. Ethanol and methanol, which are both polar solvents, are frequently utilized in the extraction of bioactive substances such as polyphenols, flavonoids, and alkaloids. In contrast, acetone, which is a solvent with non‐polar characteristics, is very suitable for the extraction of carotenoids. Conversely, hexane, also a non‐polar solvent, is the favored choice for extracting lipophilic substances such as essential oils.

There are several extraction techniques, such as maceration, percolation, and Soxhlet extraction. The process of maceration entails immersing the plant material in a solvent for a prolonged duration, hence facilitating the extraction of compounds. In contrast, percolation involves the process of solvent passage through a densely packed column containing the plant material. The Soxhlet extraction method involves the use ofspecialized equipment that enables the continuous circulation of the solvent through the plant material, therefore simplifying the extraction process.

The selection of solvent and extraction methodology has a crucial role in determining the quantity and quality of the bioactive compounds obtained. Therefore, it is crucial to optimize the extraction conditions, which include variables such as the concentration of the solvent, duration of extraction, temperature, and the ratio of solvent to plant material. These parameters collectively have a significant role in enhancing the efficiency and yield of the extraction process.

### 2.2 Ultrasound ‐Assisted Extraction (UAE)

Ultrasound‐assisted extraction (UAE) is a powerful technique used to extract various bioactive compounds from plant materials (Rivera‐Mondragón et al. 2019) and animal tissues (Bhat et al. 2022). It is a non‐destructive, environmentally friendly, and efficient method that has gained significant attention in recent years.

**FIGURE 1:**
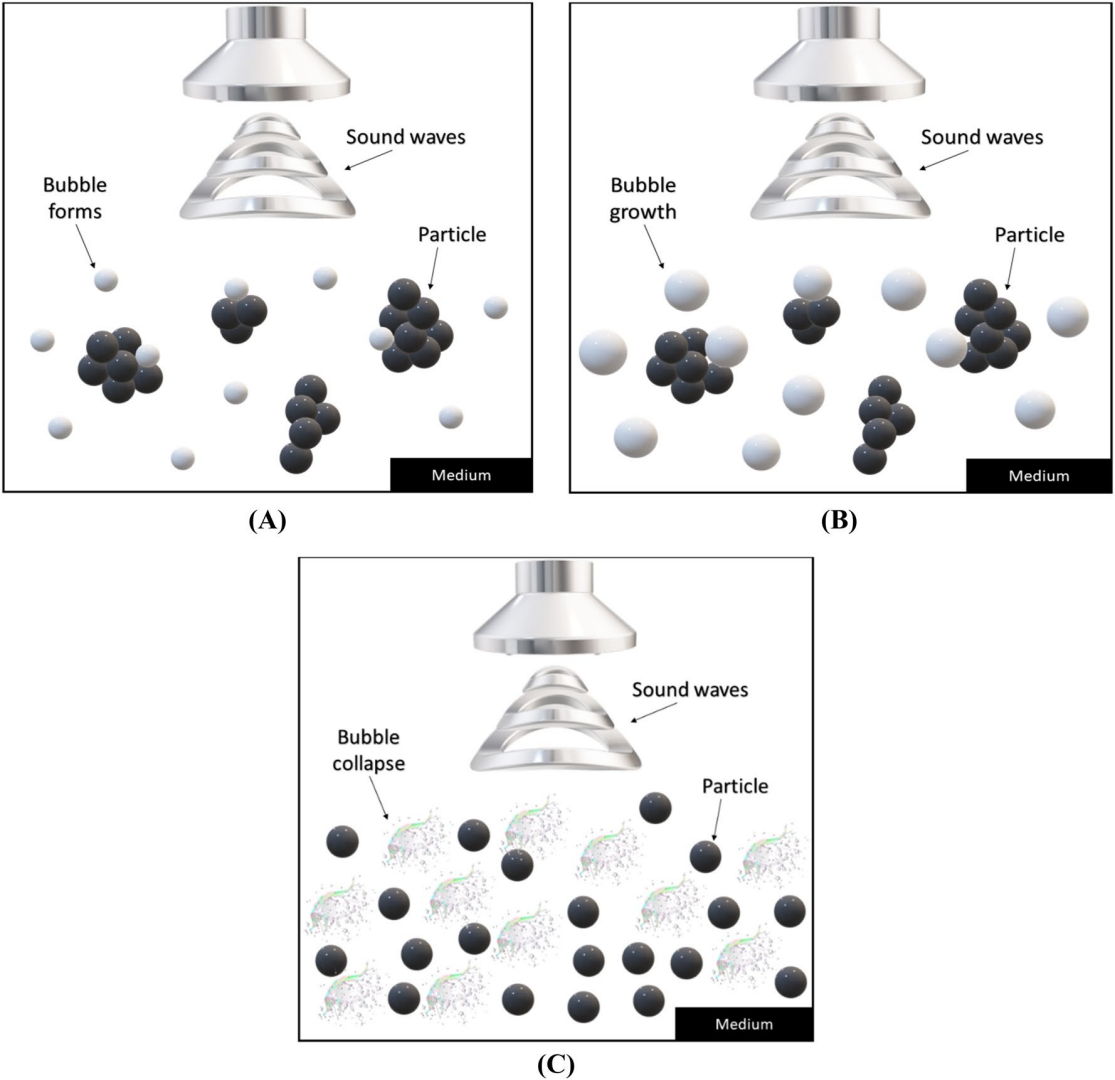
3D scheme of cavitation mechanism of action. (A) Bubbles formation by sound waves; (B) maximum growth size of bubbles; (C) bubbles burst, particles dispersion, and cell disruption occur.

UAE involves the application of high‐frequency sound waves to a liquid medium containing the sample of interest (Marić et al. 2018). The sound waves create high‐pressure zones and low‐pressure zones in the liquid, which cause cavitation bubbles to form and collapse rapidly (Sanou et al. 2023) (Figure 1). These bubbles produce intense mechanical forces that disrupt the cell walls of the sample and enhance the mass transfer of the bioactive compounds from the solid phase to the liquid phase (Kobus et al. 2022).

UAE presents numerous advantages over conventional extraction methods. First, it notably shortens extraction time, which is particularly useful for thermolabile compounds that are sensitive to heat (Dassoff and Li 2019). Additionally, UAE enhances the yield of bioactive compounds and preserves their quality by minimizing degradation and oxidation during the extraction process (Rao et al. 2021). Furthermore, UAE is a solvent‐free technique, eliminating the need for harmful solvents and reducing the environmental footprint of the extraction process (Marić et al. 2018). UAE has been effectively employed to extract various bioactive compounds, including flavonoids (Fan et al. 2022), alkaloids (Qin and Xi 2021), phenolic acids (Zhong et al. 2019), and essential oils (Kumar, Chopra, et al. 2023). Its application spans multiple industries, such as food, pharmaceuticals, cosmetics, and nutraceuticals, where it is used to extract bioactive compounds from diverse sources, as summarized in Table 1.

**TABLE 1:** Different extraction methods.

Extraction method	Sources	Conditions	Results	References
Ultrasound‐assisted extraction	Orange ( *Citrus sinensis* ) peel	Ultrasound power: 100, 250, and 400 W Ultrasound irradiation time: 5, 17.5, and 30 min Concentration of ethanol in water: 0%, 25%, and 50%	The optimum extraction conditions of bioactive orange peel compounds using UAE corresponded to the maximum tested values of ultrasonic power, extraction time, and percentage of ethanol in water	Montero‐Calderon et al. (2019)
	Custard apple	Solvent: water Power: 30 W Time: 150 s 75% duty cycle Liquid–solid ratio: 10 mL/g	Lipid extraction yield (control): 30.16% Lipid extraction yield (UAE): 33.6%	Panadare et al. (2020)
	Maidenhairtree seed	Ultrasound treatment (A): 373 W, 41 min, and 52°C Ultrasound treatment (B): 400 W, 40 min, and 50°C	Ultrasound treatment (A): 5.91% Ultrasound treatment (B): 5.89%	Wang, Xiong, and Huang (2023)
	Maize	Extraction temperature: 54.7°C Ultrasonic power: 250 W Extraction time: 42.8 min Liquid–solid ratio: 27.9 mL/g	The optimum extraction rate was 0.56%, which was more efficient than that of the simple water extraction and alcoholic sedi‐mentation method	Song, Xiong, and Huang (2023)
	*Ginkgo biloba* leaves	Ultrasound power: 288–360 W Ultrasound time: 40–50 min Liquid–solid ratio: 15–35 mL/g	The optimum yield was 5.37% at a liquid to material ratio of 30 mL/g, an ultrasonic power of 340 W and an extraction time of 50 min	Li et al. (2023)
	Bombay locusts	Ultrasonication power (100%): 750 W Amplitude: 40%, 60%, and 80% Frequency: 20 kHz Pulse mode: 5 s on‐time and 5 s off‐time. Ultrasonication time: 10, 20, and 30 min. Temperature: controlled at 25°C–35°C	60% amplitude for 20 min could be a promising condition for extracting the protein from Bombay locust with less adverse effect	Kingwascharapong et al. (2021)
	Waste biomass	Ultrasound power: 320 W Ultrasound frequency: 35 kHz Solvent: Ethanol 70% v/v Treatment (1): 60 min, and 25°C. Treatment (2): 60 min, and 50°C	Total phenolic content (mg GAE/g) Treatment (1): 13.68 Treatment (2): 19.1	Barjoveanu et al. (2020)
	*Macadamia integrifolia*	Capacity: 2.75 L Ultrasound power: 80 W Ultrasound frequency: 37 kHz Time: 5, 10, and 15 min	The ultrasound‐assisted extraction produced the extract with the greatest yield and the largest amount of phenolic content, resulting in the extract (SON) having the most effective antioxidant and anti‐skin aging activities. Best results were achieved from the 15 min treatment followed by the 5 min treatment then the 10 min treatment	Somwongin et al. (2023)

	Broiler chicken trachea	Capacity: 300 mL Probe: 25‐mm diameter cylindrical titanium alloy Solvent: water The probe was immersed into the water at a depth of 5 cm Pulse mode: on‐time 5 s and off‐time 5 s. Temperature: 25°C Ultrasound intensities: 9.80, 17.46 and 27.56 W·cm^−2^ Ultrasound time: 10, 20 and 30 min	Conventional extraction by pepsin resulted in 3.1% yield. Trachea collagen yield was increased to 6.28%, after ultrasound with an intensity of 17.46 W·cm^−2^ for an exposure time of 20 min	Kaewbangkerd et al. (2023)
*Tenebrio molitor* larvae	Suspension pH: 10 Temperature: 25°C Ultrasound power: 4 W/cm^−2^ Ultrasound frequency: 28 kHz Ultrasound time: 0, 10, 20, 30, 40, and 50 min	The treatment with modified molecular structures and higher in vitro digestibility which obtained with an ultrasound‐assisted extraction processing time (30 min) and could act as a novel and sustainable protein source that could be potentially applied to many food formulations	Zhang et al. (2023)
Microwave‐assisted extraction	Aromatic plants	Optimum conditions were studied with the following solvents: 20% (v/v) aqueous methanol solution 80% (v/v) aqueous methanol solution	The optimum extraction conditions were: For chlorogenic acid, 50% microwave power, 30 s microwave irradiation, 1:20 (g/mL) ratio of solid to liquid, and 20% (v/v) aqueous methanol solution as extractant For geniposidic acid, 50% microwave power, 40 s microwave irradiation, 1:20 (g/mL) ratio of solid to liquid, and 80% aqueous methanol solution	Zhang et al. (2011)
	Grape pomace	Microwave frequency: 2450 MHz pH values: 1, 2 and 3. Solid–liquid ratio: 1:10 (w/v) Radiation power: 280, 420 and 560 W. Exposure time: 60, 90 and 120 s. Temperature: 25°C	The optimal conditions for pectin extraction were 560 W, pH of 1.8 for 120 s	Spinei and Oroian (2022)
	Ginger	Microwave frequency: 2.45 × 109 Hz Microwave power: 400, 600, and 800 W. Reaction time: 1, 3, and 5 min	The optimal conditions were microwave power of 400 W and an extraction time of 1 min	Utama‐ang et al. (2021)
	Berberis roots	Microwave power: 100 and 300 W. Irradiation time: 2 and 5 min. Sample‐to‐solvent ratio: 20 and 50 g/mL. Solvent pH: 2.5 and 6. Solvent concentration: 20% and 80%	The optimum conditions were: For Berberine and Palmatine: 100 W, 2 min, 50 g/mL, pH 2.5, and 80% concentration. For total phenols: 300 W, 5 min, 50 g/mL, pH 2.5, and 20% concentration	Belwal et al. (2020)
	Swertia chirata	Solvents: ethanol, aqueous ethanol (25%–75% v/v) and water. 1 g powdered leaves were heated at 75°C temperature with 50 mL of different solvents for 1 h. Microwave power: 450 W. Time: 2 min	Maximum Mangiferin quantity was observed with 50% ethanol	Kaur et al. (2021)

	*Centella asiatica* (L.)	Ethanol percentage: 40%–80%. Microwave power: 100–200 W. Extraction time 5–10 min	Optimal conditions: 80% ethanol, power 100 watts, 7.5 min	Thong‐on et al. (2021)
	Trigonella foenum graceum seed	Sample‐solvent ratio: 1:5 (w/v). Solvents: Acetone, ethanol, hexane and petroleum ether. Solvent concentration: 40%, 60%, 80% and 100%. Extraction time: 1.5, 3.0, 4.5 and 6.0 min. Fixed power of 180 W	Optimum Diosgenin extraction conditions were: Solvent: Ethanol. Solvent concentration: 80%. Extraction time: 6 min	Arya and Kumar (2021)
	Heterophyllaea pustulata	Plant material was subjected to one hour extraction with 20 mL of hexane to eliminate chlorophylls and fatty components. The remaining vegetable material of each sample (stems and leaves) was extracted at a constant temperature of 50 C (maximum temperature of the ultrasonic bath) during 15, 30, 45 and 60 min, using 20 mL of benzene. Each sample was then extracted with ethyl acetate, after being treated with benzene for 1 h. The parameters (temperature, amount of solvent and extraction time) applied in the ethyl acetate extractions were the same as those for benzene. Irradiation power: 450, 630 and 900 W. Time: 15, 30 and 60 min	Solvent: Ethyl acetate. Irradiation power: 900 W. Time: 15 min	Barrera Vázquez et al. (2014)
Wet oleaginous yeast biomass	Solvent: Chloroform and methanol 2:1 (v/v) Irradiation power: 600 W during the heating process and 500 W during the holding process. Reaction temperature: 60°C–100°C. Ratio of volume of chloroform/methanol to the weight of wet oleaginous biomass: 2:1 to 8:1. Reaction time: 2–15 min	Optimum extraction conditions were: Reaction temperature: 90°C. Ratio of volume of chloroform/methanol to the weight of wet oleaginous biomass: 6:1. Reaction time: When the time was raised from 10 to 15 min, the lipid content (wt/wt; %) increased with a comparatively low rate compared to the first 10 min of reaction and this indicates the accomplishment of the extraction process	Patel et al. (2019)
High‐pressure homogenization	Coconut mesocarp	Rotational speed: 11200 g Time: 2.5, 5, 7.5 and 10 min	Optimum extraction conditions were 11200 g for 10 min	Yang et al. (2021)
	Okara	Shear speed: 2800, 11200, and 25200 g. Shear time: 10 min	Optimum conditions were 25200 g for 10 min	Liu, Yi, et al. (2021)
	*Ruta chalepensis*	Orifice diameter: 150 μm at 100 MPa for up to 10 passes. After each pass, the suspensions were cooled down in a tube‐in‐tube heat exchanger set at 5°C, to ensure that the product temperature was always below 25°C	When comparing the effect of maceration and high‐pressure homogenization on the recovery of quercetin and rutin, it can be observed that, in butanol, rutin increased more than 29.8% and in ethyl acetate, quercetin increased more than 452.7%	Gali et al. (2020)

	Algal cell	Pressure applied: varied between 10,000 (68.948 MPa) to 45,000 PSI (310.264 MPa). Average throughput: 50 mL/min. Orifice diameters: 100, 130, and 190 μm. Temperature 25°C–70°C. Number of passes: 1–6 passes	High‐pressure homogenization proved to be an effective technique to rupture cell walls. An initial partial factorial design showed that the outlet diameter of the nozzle does not significantly affect the degree of cell wall lyses. The most significant parameters were the pressure differential across the nozzle and the number of passes through the homogenizer. The concentration of the algal culture and the level of stress of the culture were also significant but to a lesser degree	Samarasinghe et al. (2012)
	Microalgae Parachlorella kessleri	Average throughput: 10 L/h. Homogenizing pressure: fixed in the range of 400 to 1200 bar (1 bar = 105 Pa). Number of passes: varied from 1 to 10. Initial temperature of suspensions before procedure was 22°C Temperature elevation after treatment never exceeded 30°C. Before each pass through the homogenizer, the suspension was cooled to 22°C	Compared to ultrasonication treatment only, the preliminary ultrasonication of more concentrated suspensions followed by high‐pressure homogenization of suspensions allowed increasing the extraction efficiency and decreasing the energy consumptions	Zhang et al. (2019)
	Soybean okara	Flow rate: 10 L/h. Aliquots of 150 mL of okara dispersion at 20°C were subjected to single‐pass at a pressure of 50, 100 and 150 MPa and at 5 passes at 150 MPa. Sample temperature was measured immediately after treatment. After treatments, all the samples were cooled at room temperature (20°C) by immersion into an ice bath under gentle mixing	High‐pressure homogenization can be used as an efficient tool to induce a progressive disruption of okara native structure, leading to the release of entrapped proteins and soluble fibers. High‐pressure homogenization might thus be applied as a pre‐treatment to favor extraction of proteins and fibers, allowing okara by‐product to be turned into added‐value ingredients for the food industry	Fayaz et al. (2019)
	Peanuts	Defatted peanut flour was dispersed into distilled water at the ratio of 1:10, adjusted to pH 8.5 with 1 M NaOH, and finally stirred using a magnetic stirrer for 1 h. The resultant dispersion was divided further into three aliquots. One aliquot applied to high‐pressure homogenization under 0.1 MPa was used as control. The other two aliquots were subjected to the high‐pressure homogenization under 40 or 80 MPa	High‐pressure homogenization treatment increased the extraction yield and hydrolysis degree of PPI. Gel filtration chromatography showed that the high‐pressure homogenization treatment increased the enzymatic hydrolysis of PPI, as indicated by smaller peptides. High‐pressure homogenization treatment increased reducing power, DPPH radical scavenging and hydroxyl radical scavenging activities of the PPI hydrolysates. High‐pressure homogenization treatments at higher pressure (up to 400 MPa) proved to improve food functional properties	Dong et al. (2011)
Microalgae *Chlorella vulgaris*	Orifice diameter: 100 μm. Pressure: 150 MPa Flow rate: 155 mL/min. Number of passes: 1–10. To prevent excessive heating, after each pass, the suspensions were cooled at 25°C by passing through a tube‐in‐tube heat exchanger, located downstream of the orifice valve	High‐pressure homogenization was able to disrupt completely the microalgae cells, favoring an instantaneous and efficient release of all the intracellular material, including a large amount of proteins. Release was 10.3‐fold higher than by PEF. Despite the higher extraction efficiency, the formation of large amounts of finely sized cell debris by high‐pressure homogenization significantly complicates any downstream separation process	Carullo et al. (2018)
Enzyme‐assisted extraction	Brown seaweeds	pH: 4.5–8 Temperature: 40°C–60°C Buffer used: 0.1 M acetate and 0.1 M phosphate	The yield of the enzymatic extracts of different seaweeds ranged from 30.3 ± 1.3 to 92.9% ± 2.2% while the yield of water extracts ranged from 18.7% ± 0.3% to 50.8% ± 1.3%	Habeebullah et al. (2020)
	Sweet cherry ( *Prunus avium* L.) pomace	Enzymes quantity: 1–120 μL/g pH: 3.0–8.0 Temperature: 30°C–70°C Time: 30–300 min	Optimal extraction conditions for extracting non‐extractable polysaccharides (NEPs) using enzyme‐assisted extraction (EAE) with Depol, Promod, and Pectinase enzymes were found to be at a temperature of 70°C and a pH of 10.0. The ideal extraction time was 40 min for both Depol and Promod enzymes, whereas 18.4 min was optimal for Pectinase enzyme. The optimum enzyme concentrations were 140 μL of Promod per gram of sample, 90 μL of Depol per gram of sample, and 2 μL of Pectinase per gram of sample	Domínguez‐Rodríguez et al. (2021)
	Citrus fiber	Enzymes quantity: 0.45% pH: 4.5–6.5 Temperature: 50°C Time: 120 min Liquid to substrate ratio: 1:20	Among the modification methods in this research, the combination of xylanase treatment and water media ball milling (WMBM) was the most effective way to modify citrus fiber. The combination of xylanase hydrolysis and dry micronization had few improvements of citrus fiber's properties compared to xylanase‐WMBM treatment	Song et al. (2021)
	Banana peel	Enzymes quantity: 5FPU/mL pH: 6.0–7.0 Temperature: 50°C Time: 120 h Liquid to substrate ratio: 1:20	The enzymatic hydrolysis was superior to dilute‐acid hydrolysis by providing higher levels of soluble fibers of cellodextrins and water‐soluble cellulose without the occurrence of any degraded byproducts	Phirom‐on and Apiraksakorn (2021)
	Oat flours	Enzymes quantity: 0.01% pH: 5.0–9.0 Temperature: 100°C Time: 15–75 min Liquid to substrate ratio: 1:5	The free and conjugated phenolic fractions markedly increased, resulted in a modification in the proportion of the phenolic fractions and the promotion of antioxidant activities in oats	Chen et al. (2020)
	Coffee coproducts (CCP)	Enzymes quantity: 5 − 15 U pH: 5.0–6.0 Temperature: 50°C Time: 30–20 min Liquid to substrate ratio: 1:25	Enzymatic reaction with at least 5 U of cellulase for 30 min was sufficient to maximize the technological properties of CCP within conditions. These results imply the potential to obtain sustainable food ingredients from modified CCP, bringing about improved technological properties and significant antioxidant status	Belmiro et al. (2021)
	Blackcurrant	Enzymes quantity: 108 U/g pH: 5.0–6.0 Temperature: 60°C Time: 10–90 min Liquid to substrate ratio: 0.1:15 and 0.2:15	No significant differences between the amount of total anthocyanins extracted by ultrasound‐assisted extraction and enzyme‐assisted extraction	José Aliaño González et al. (2022)
	Eggplant ( *Solanum melongena* L.) peel	Temperature: 35°C–60°C Time: 1–4.50 h Enzyme concentration: 5%–15% E/S	The optimum condition of EAE parameters (Temperature 37.32°C, Enzyme concentration 5% and Time 1 h) was achieved to attain maximum response properties. The predicted optimum value of each response was 71.45% for TY, 578.665 (mg C3G/l) for TAC, 2040.87 (mg GAE/L) for TPC, 79.91% for DPPH and 29.90 (mmol AAE/100 g) for FRAP. Enzyme‐assisted extraction was clearly recognized as an effective way to extract bioactives from eggplant peel namely, total anthocyanins, phenolics, and antioxidant activity	Amulya and ul Islam (2023)

Despite its advantages, the application of UAE requires careful optimization of the extraction conditions (Barjoveanu et al. 2020), such as the ultrasound frequency, power, and duration (Thong‐on et al. 2021), as well as the sample‐to‐solvent ratio and temperature (Kaur et al. 2021). The selection of appropriate parameters depends on the nature of the sample and the target compound.

Response surface methodology (RSM) is a powerful statistical tool that can significantly enhance UAE processes by optimizing critical variables and improving extraction efficiency. UAE is widely used for extracting bioactive compounds, such as polyphenols, flavonoids, and essential oils, from plant materials. The ultrasound waves create cavitation bubbles that disrupt the plant cell walls, increasing solvent penetration and facilitating the release of these compounds. However, the efficiency of UAE depends on several key parameters, including ultrasound power, extraction time, solvent concentration, temperature, and solid‐to‐liquid ratio. This is where RSM plays a crucial role.

RSM uses a mathematical and statistical approach to model and analyze the interactions between multiple factors that influence the UAE process. It allows researchers to design experiments systematically, enabling them to explore the relationships between variables and determine the optimal conditions for maximum extraction yield. By applying RSM, the extraction process can be fine‐tuned to achieve the best combination of ultrasound power, temperature, solvent type, and extraction time, minimizing the need for trial‐and‐error methods.

One of the main advantages of using RSM is its ability to account for the synergistic or antagonistic effects between different parameters. For example, higher ultrasound power might increase the extraction yield up to a certain point, but excessive power could lead to degradation of sensitive compounds. RSM helps identify these critical thresholds, ensuring the integrity of the bioactive compounds is maintained while maximizing efficiency.

Additionally, RSM reduces the number of experimental runs required to optimize the UAE process, saving time and resources. It provides a visual representation of how each factor affects the extraction through response surface plots, making it easier to predict outcomes and make informed decisions. Overall, integrating RSM into UAE processes leads to improved efficiency, higher yields, and enhanced quality of extracted bioactive compounds.

UAE is an innovative technique for extracting bioactive compounds from various sources. It presents numerous advantages over conventional methods, such as shorter extraction times, enhanced yields and quality of bioactive compounds, and a lower environmental impact. Its broad use across various industries underscores its potential as a valuable tool for obtaining high‐value compounds efficiently.

### 2.3 Microwave‐Assisted Extraction (MAE)

**FIGURE 2:**
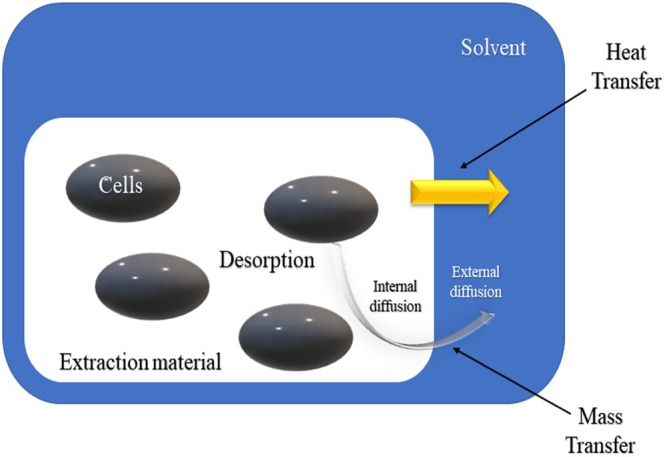
3D scheme of microwave‐assisted extraction.

Microwave extraction has gained significant attention for its effectiveness and efficiency in extracting bioactive compounds from plant materials (Lee et al. 2016). This technique utilizes electromagnetic waves, typically within the 300 MHz to 300 GHz frequency range, to heat and extract compounds from plant matrices (Belwal et al. 2017). The process works by inducing an interaction between these electromagnetic waves and the polar molecules present in both the solvent and plant material (Pellati et al. 2013). This interaction transforms electromagnetic energy into thermal energy, generating heat that facilitates the extraction of bioactive compounds (Hoang et al. 2007; Wang et al. 2017), as illustrated in Figure 2.

The efficiency of microwave extraction is influenced by several key factors, including the power and frequency of the microwave radiation, the solvent used, and the characteristics of the plant material (Belwal et al. 2018). High power and frequency enhance extraction efficiency by promoting rapid and uniform heating, whereas low power and frequency may result in incomplete extraction and potential thermal degradation of the compounds (Jacotet‐Navarro et al. 2016; Zill‐e‐Huma et al. 2011). The choice of solvent is critical for both extraction efficiency and selectivity (Kaur et al. 2020). Polar solvents like water, ethanol, and methanol are preferred in microwave extraction due to their strong dielectric properties, which enable effective interaction with the polar molecules in the plant matrix (Assefa et al. 2019). In contrast, non‐polar solvents such as hexane and ethyl acetate are less commonly used because of their lower dielectric properties and limited ability to dissolve polar compounds (Kaur et al. 2020).

The characteristics of plant material, including particle size, moisture content, and chemical composition, can significantly influence extraction efficiency (Zou et al. 2013). Finer particles increase surface area, enhancing mass transfer between the solvent and plant matrix. However, high moisture content can cause superheating, leading to thermal degradation of the extracted compounds (Khakpour et al. 2023). Microwave extraction offers distinct advantages over conventional methods, making it a promising alternative for extracting bioactive compounds from plants (Belwal et al. 2017; Pellati et al. 2013). One key advantage is the reduced processing time; microwave extraction is rapid and efficient, cutting extraction times significantly compared to traditional methods like Soxhlet and maceration (Armenta et al. 2017; Jo et al. 2020; Phoboo et al. 2011). This efficiency is due to the uniform heating generated by electromagnetic waves, which accelerates mass transfer between the solvent and plant matrix (Kellogg et al. 2017; Wang, Hu, et al. 2016). Moreover, microwave extraction often achieves higher yields than conventional techniques by penetrating deeper into the plant matrix and accessing compounds that are otherwise difficult to extract (H.‐F. Zhang et al. 2011). Electromagnetic waves induce selective heating of polar molecules, facilitating the release of target compounds (Wang, Ding, and Ren 2016).

#### 2.3.1 Improved Selectivity

Microwave extraction can selectively extract specific compounds from the plant matrix by using solvents with specific properties and optimizing the extraction conditions (Maran and Prakash 2015). This is because microwave extraction can selectively heat the polar molecules in the plant matrix and minimize the extraction of unwanted compounds such as fats, waxes, and chlorophyll (Rahmani et al. 2020).

#### 2.3.2 Cost‐Effective

Microwave extraction can be cost‐effective compared to conventional methods due to its reduced processing time, lower solvent consumption, and higher extraction yields (Pangestu et al. 2020). This can lead to significant savings in terms of energy, labor, and equipment costs.

#### 2.3.3 Environmentally Friendly

Microwave extraction can be a more environmentally friendly option compared to conventional methods that use large amounts of solvents and generate high volumes of waste (Karbuz and Tugrul 2021). Microwave extraction can use smaller amounts of solvents and generate less waste, leading to a more sustainable extraction process (Rodsamran and Sothornvit 2019; Thu Dao et al. 2021).

The efficiency of microwave extraction is influenced by several factors, including the power and frequency of the microwave radiation, the nature of the solvent used, and the characteristics of the plant material (Table 1). Optimization of these factors is crucial for achieving maximum extraction efficiency and selectivity.

Preparing samples with a high‐speed homogenizer before microwave‐assisted extraction (MAE) can significantly enhance the efficiency and yield of bioactive compound extraction. A high‐speed homogenizer is used to pre‐treat the sample by breaking down plant or biological tissue into smaller, more uniform particles, thereby increasing the surface area exposed to the extraction solvent. This preparatory step plays a crucial role in improving mass transfer and facilitating the release of target compounds, such as polyphenols, flavonoids, and essential oils, during the MAE process.

When samples are homogenized at high speeds, the cell walls and other structural barriers are disrupted, allowing easier access to intracellular components. This breakdown of cellular structures ensures that the solvent penetrates more effectively, leading to enhanced interaction between the solvent and the target compounds. As a result, the homogenized samples respond better to microwave energy, which heats the solvent and sample more uniformly. The uniform heating during MAE further accelerates the extraction process by causing localized cell rupture and enhancing solubility and diffusion rates of bioactive compounds into the solvent.

The synergy between homogenization and MAE is particularly important for hard‐to‐extract compounds or when dealing with tough plant matrices. The increased surface area and reduced particle size after homogenization allow the microwave energy to act more effectively, reducing the time needed for extraction while increasing the yield. This not only improves extraction efficiency but also helps to preserve the integrity of heat‐sensitive compounds that might degrade with prolonged microwave exposure.

Moreover, high‐speed homogenization as a pre‐treatment step minimizes the need for excessive solvent use, leading to a more sustainable and environmentally friendly extraction process. In combination, these techniques offer a powerful approach for maximizing the extraction of valuable bioactive compounds in industries like food, pharmaceuticals, and cosmetics, while reducing energy consumption and processing time.

### 2.4 High‐Pressure Homogenization

High‐pressure homogenization (HPH) is a green extraction method that has gained increasing attention in recent years due to its ability to extract bioactive compounds from natural sources without the use of harsh chemicals or solvents (Tan and Kerr 2015). This technique offers several advantages over traditional extraction methods, such as reduced energy consumption, lower environmental impact, and the ability to produce high‐quality products (Patrignani et al. 2020).

**FIGURE 3:**
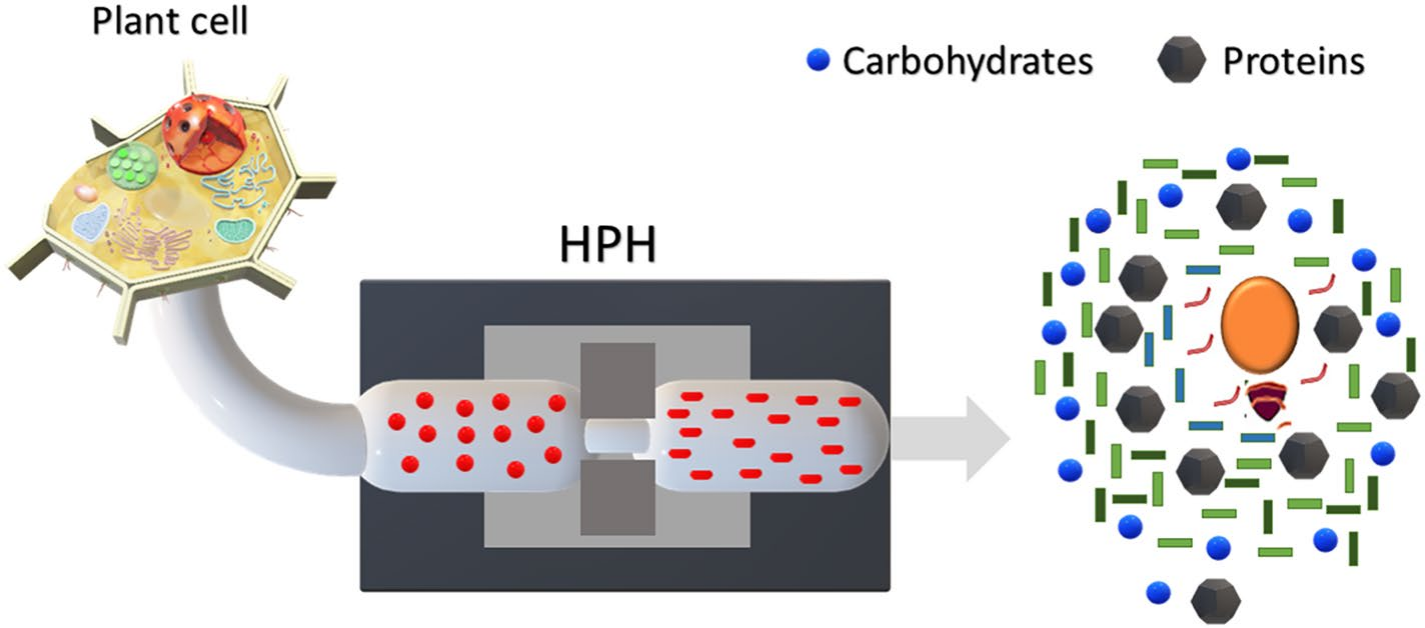
3D scheme of high‐pressure homogenization extraction.

HPH is a green extraction method that applies intense mechanical forces to a mixture of two immiscible liquids, such as oil and water, to extract bioactive compounds from natural sources (Zhu et al. 2016) (Figure 3). The mechanism of action behind HPH involves a series of physical phenomena that occur during the homogenization process.

The first step in the mechanism of action is the creation of high‐energy zones within the homogenizer. As the mixture passes through a narrow gap at high pressure, intense turbulence and shear forces are generated (Bevilacqua et al. 2019). This leads to the creation of a large interfacial area between the two immiscible liquids and the breakup of larger droplets into smaller ones (Georget et al. 2014).

The second step in the mechanism of action involves the release of intracellular compounds from natural sources. HPH is highly effective at disrupting plant cells and releasing trapped intracellular compounds, including phytochemicals, vitamins, and minerals (Zhu et al. 2016). This is achieved by applying high pressure, which breaks down the cell walls and facilitates the release of these bioactive compounds. The resulting extracts are typically rich in valuable compounds and have a wide range of applications in industries such as food, cosmetics, and nutraceuticals (Saricaoglu et al. 2019; Wang et al. 2018; Wu et al. 2020).

The third step in the mechanism of action is the preservation of bioactive compounds during the extraction process (Zhou 2021). Unlike traditional extraction methods that use high temperatures and harsh chemicals, HPH operates at low temperatures and without the use of solvents or chemicals, which reduces the risk of degradation of bioactive compounds. The short processing time and the ability to control the temperature during the extraction process also minimize the risk of degradation of bioactive compounds (Quan et al. 2020; Sentandreu et al. 2020).

HPH is an environmentally friendly extraction method that offers several advantages over traditional techniques. One major benefit of HPH is its efficiency in extracting bioactive compounds from natural sources such as plants, fruits, and vegetables (Betoret et al. 2017). The process uses high pressure to disrupt plant cells and release intracellular compounds, resulting in extracts that are typically rich in phytochemicals, vitamins, and minerals (Martínez‐Monteagudo et al. 2017). These extracts have diverse applications, including in food, cosmetics, and nutraceuticals (Zamora and Guamis 2015), as outlined in Table 1.

Additionally, HPH excels in producing high‐quality products with minimal degradation of bioactive compounds (Sharabi et al. 2018). This advantage stems from its short processing times and controlled temperatures, which help preserve the integrity of the compounds. Unlike traditional methods that often require high temperatures and harsh chemicals, HPH operates at lower temperatures and without solvents or chemicals, thus reducing the risk of degradation (Karacam et al. 2015; Moscovici Joubran et al. 2019; Saricaoglu et al. 2019; Silva et al. 2010; Suárez‐Jacobo et al. 2011; Wellala et al. 2020).

However, HPH is a green extraction method that offers several advantages over traditional extraction methods. It provides efficient extraction of bioactive compounds from natural sources with minimal degradation and has a low environmental impact due to the absence of solvents or chemicals. As the demand for sustainable and environmentally friendly extraction methods continues to grow, HPH is expected to become an increasingly popular choice in various industries.

### 2.5 Enzyme‐Assisted Extraction (EAE)

Enzyme‐assisted extraction (EAE) is an advanced technique used for extracting bioactive compounds from plant‐based foods (see Table 1). This method involves the use of specific enzymes that break down the cell walls of plant materials, thereby facilitating the release of bioactive compounds. EAE offers several advantages over traditional extraction methods, such as increased extraction efficiency, reduced solvent consumption, and the ability to selectively target and extract desired compounds (Sharif et al. 2023).

The mechanism of EAE revolves around the utilization of tailor‐made enzymes that act upon the cell walls of plant material. Enzymes, which are biological catalysts, accelerate chemical reactions without being consumed in the process. In the context of EAE, enzymes target the complex polysaccharides and lignin constituting the cell walls, causing their breakdown (Islam et al. 2023).

The breakdown of cell walls by enzymes enables the facile release of bioactive compounds. Once the cell walls are dismantled, the bioactive compounds can freely diffuse into the extraction solvent, typically water or a blend of water and a polar organic solvent. The EAE process can be further refined by adjusting parameters such as enzyme concentration, reaction time, and temperature (Sanjeewa et al. 2023).

The efficiency of EAE can be enhanced by employing a combination of enzymes that target different components of the plant cell wall. For instance, enzymes targeting cellulose can be employed alongside those targeting hemicellulose or lignin, resulting in a comprehensive breakdown of cell walls and elevated extraction efficiency (Islam et al. 2023).

Notably, one of the primary advantages of EAE lies in its capability to selectively extract specific bioactive compounds. Enzymes can be selected based on their capacity to target particular compounds, leading to a more precise and efficient extraction process. Furthermore, EAE facilitates the extraction of bioactive compounds that may pose challenges when using traditional methods (Freitas et al. 2023).

To summarize, the mechanism of EAE involves the utilization of specific enzymes to dismantle the cell walls of plant material, facilitating the release of bioactive compounds. EAE offers notable advantages over conventional extraction methods, including heightened extraction efficiency, reduced solvent usage, and the ability to selectively extract desired compounds. The efficiency of EAE can be further refined by adjusting enzyme concentration, reaction time, and temperature, as well as employing a combination of enzymes to target different components of the plant cell wall.

## 3 Isolation and Purification Techniques of Bioactive Compounds from Plant‐Based Foods

### 3.1 Solid‐Phase Extraction (SPE)

Solid‐phase extraction (SPE) is a highly effective technique for the extraction and purification of bioactive compounds from plant‐based foods. It involves the use of solid adsorbents to selectively retain target compounds, allowing for their isolation from complex matrices (Attallah et al. 2023).

SPE offers several advantages that make it a valuable tool in the field of natural product research. Firstly, it provides excellent selectivity, enabling the isolation of specific bioactive compounds of interest while minimizing interference from other components present in the sample. This selectivity is achieved by carefully choosing the appropriate adsorbent material that exhibits affinity toward the target compounds (Kumar, Kaushik, et al. 2023).

Another advantage of SPE is its versatility. It can be tailored to suit different sample types and compound classes. By selecting the appropriate adsorbent, sorbent bed size, and elution solvent, researchers can optimize the extraction conditions and achieve high recovery yields for a wide range of bioactive compounds (da Silva et al. 2020).

The method is also known for its efficiency. SPE typically requires minimal solvent consumption compared to other extraction techniques, which is both cost‐effective and environmentally friendly. Additionally, the use of solid‐phase sorbents eliminates the need for laborious filtration steps, resulting in time savings and simplified sample preparation (Patrice Didion et al. 2023).

SPE is widely utilized in natural product research to isolate and purify various bioactive compounds, including phenolic compounds, flavonoids, alkaloids, and terpenoids. These compounds possess diverse biological activities and are of great interest in fields such as pharmaceuticals, nutraceuticals, and functional foods (Mir‐Cerdà et al. 2023).

To perform SPE, the sample is initially passed through a solid‐phase cartridge or disk containing the appropriate adsorbent material. The target compounds selectively interact with the sorbent, while undesirable matrix components are washed away. Afterward, the retained compounds are eluted using a suitable solvent, resulting in their isolation and concentration for subsequent analysis (Waseem et al. 2023).

Thus, SPE is a valuable technique for the extraction and purification of bioactive compounds from plant‐based foods. Its selectivity, versatility, efficiency, and broad application make it an essential tool in natural product research. By utilizing SPE, researchers can effectively isolate and purify bioactive compounds, leading to a deeper understanding of their properties and potential applications in various industries.

### 3.2 Centrifugal Partition Chromatography (CPC)

Centrifugal partition chromatography (CPC) is a sophisticated chromatographic technique that has garnered significant interest in natural product research for extracting, isolating, and purifying bioactive compounds from plant‐based sources. It offers several advantages over traditional chromatographic methods, making it a valuable tool for discovering novel bioactive compounds (Métoyer et al. 2024). CPC operates on the principle of liquid–liquid partitioning, utilizing a two‐phase solvent system. In this method, the sample mixture is introduced into a column filled with a stationary phase, typically a high‐density liquid, while a low‐density liquid serves as the mobile phase. Centrifugal force drives the formation of a biphasic system, enabling the partitioning and separation of target compounds based on their partition coefficients (Kiene et al. 2023).

One of the primary advantages of CPC is its high loading capacity. The biphasic nature of the system enables the loading of large sample volumes, making it ideal for processing complex plant extracts with high concentrations of bioactive compounds. This feature significantly reduces the need for extensive sample preparation, saving time and resources (Michel et al. 2024).

CPC also offers excellent separation efficiency. The continuous recycling of the mobile phase allows for multiple partitioning events, enhancing the resolution of target compounds. Furthermore, CPC can be easily scaled up, enabling the purification of larger quantities of bioactive compounds for further characterization and bioassays (Nunes et al. 2023).

Another notable advantage of CPC is its ability to operate in an isocratic mode, meaning that the mobile phase composition remains constant throughout the separation. This eliminates the need for gradient elution, simplifying method development and allowing for better reproducibility and robustness (Nakonieczna et al. 2024).

CPC has demonstrated exceptional selectivity, especially for structurally diverse compounds. By adjusting the composition of the stationary and mobile phases, researchers can fine‐tune the separation parameters and achieve high‐purity fractions of bioactive compounds. This selectivity makes CPC particularly valuable in the isolation of minor components from complex plant matrices (Pajot et al. 2023).

Furthermore, CPC is a versatile technique that can be employed with a wide range of solvent systems and column configurations. This flexibility enables researchers to tailor the method to specific compound classes or sample types, enhancing its applicability in various natural product research areas (de Souza et al. 2023).

CPC is a powerful chromatographic technique for the extraction, isolation, and purification of bioactive compounds from plant‐based sources. Its high loading capacity, separation efficiency, selectivity, and versatility make it an attractive alternative to conventional chromatographic methods. By harnessing the capabilities of CPC, researchers can expedite the discovery and development of novel bioactive compounds from natural sources, opening doors to potential applications in pharmaceuticals, nutraceuticals, and other industries.

### 3.3 Preparative Liquid Chromatography (PLC)

Preparative liquid chromatography (PLC) is a powerful technique used for the purification and isolation of bioactive compounds from plant‐based sources. It is a scaled‐up version of analytical liquid chromatography, specifically designed to handle larger sample volumes and produce purified fractions suitable for further analysis and applications (Shao et al. 2020).

PLC operates on the same principles as analytical liquid chromatography, involving the separation of compounds based on their differential interactions with a stationary phase and a mobile phase. However, PLC utilizes larger columns packed with specialized stationary phases to accommodate higher sample loads and achieve higher purification yields (Li et al. 2021).

One of the key advantages of PLC is its ability to handle complex mixtures present in plant extracts. By using appropriate stationary phases and optimizing separation conditions, PLC enables the efficient isolation of target compounds from intricate matrices, which may contain a wide range of co‐extracted compounds. This capability is particularly important in natural product research, where the purification of bioactive compounds is often challenging due to their low abundance or presence alongside interfering compounds (Guiochon 2002).

PLC also offers exceptional resolution and purification power. With its larger column dimensions and increased sample load, PLC allows for improved separation of closely related compounds and removal of impurities. This high resolution is crucial for obtaining highly pure fractions of bioactive compounds, which is essential for subsequent characterization and biological evaluation (Sun et al. 2023).

Furthermore, PLC provides flexibility in terms of solvent selection and gradient elution profiles. This flexibility enables researchers to fine‐tune separation conditions, optimize purification strategies, and adapt the method to different compound classes or sample types. By carefully adjusting the mobile phase composition, flow rate, and gradient profile, PLC can achieve high selectivity and purification efficiency for a wide range of bioactive compounds (Wang, Mei, et al. 2023).

PLC is a versatile technique that can be combined with various detection methods, such as UV–visible, mass spectrometry, or refractive index detection, to monitor the elution of target compounds in real‐time. This allows for efficient fraction collection, ensuring the isolation of pure compounds and minimizing sample loss (Yao et al. 2024).

Therefore, PLC is a valuable technique in natural product research for the purification and isolation of bioactive compounds from plant‐based sources. Its ability to handle complex mixtures, provide high‐resolution separation, and offer flexibility in method development makes it an essential tool in the quest for novel bioactive compounds. By harnessing the capabilities of PLC, researchers can advance their understanding of plant‐derived compounds and unlock their potential applications in various fields, including pharmaceuticals, nutraceuticals, and functional foods.

### 3.4 UV–Visible Spectroscopy

UV–Visible spectroscopy is a widely used analytical technique in various scientific disciplines, including chemistry, biochemistry, and material science. It involves the measurement of the absorption or transmission of ultraviolet (UV) and visible light by a sample, providing valuable information about its electronic structure and the presence of chromophores (Rawat and Garg 2021).

One of the key advantages of UV–Visible spectroscopy is its simplicity and accessibility. The instrumentation is relatively straightforward, consisting of a UV–Visible spectrophotometer that emits a broad range of UV and visible light wavelengths. The sample is typically placed in a transparent cuvette, and the absorbance or transmittance of light is measured, allowing for quantitative analysis.

UV–Visible spectroscopy is particularly effective for analyzing compounds with conjugated pi‐electron systems, such as organic compounds and transition metal complexes. These compounds exhibit characteristic absorption bands in the UV and visible regions due to electronic transitions between energy levels. By studying the absorption spectra, valuable information about the electronic properties, concentration, and purity of the sample can be obtained (Mir‐Cerdà et al. 2023).

The technique offers excellent sensitivity, allowing for the detection of compounds at low concentrations. This makes UV–Visible spectroscopy useful for quantitative analysis, such as determining the concentration of a compound in a solution or monitoring chemical reactions in real time (Liu, Wang, et al. 2021).

UV–Visible spectroscopy is also employed for qualitative analysis and identification of compounds. Each compound has a unique absorption spectrum, often characterized by distinct peak positions and intensities. By comparing the absorption spectrum of an unknown compound with reference spectra or databases, the compound can be identified or classified (Melo et al. 2021; Ramos‐Escudero et al. 2021; Savitharani et al. 2024; Sirijan et al. 2020).

Furthermore, UV–Visible spectroscopy is a non‐destructive technique that requires minimal sample preparation. It can be used for the analysis of a wide range of sample types, including liquids, solids, and gases. This versatility makes it a valuable tool in various scientific fields, such as pharmaceutical analysis, environmental monitoring, and quality control (Shrinet et al. 2021).

UV–Visible spectroscopy can be utilized for kinetic studies, allowing the monitoring of chemical reactions in real time. By following changes in absorbance over time, reaction rates, reaction mechanisms, and the influence of various factors on the reaction can be investigated (Souza et al. 2021).

Thus, UV–Visible spectroscopy is a versatile and widely used analytical technique for the analysis of compounds with conjugated pi‐electron systems. Its simplicity, accessibility, sensitivity, and non‐destructive nature make it a valuable tool for qualitative and quantitative analysis in various scientific disciplines. By harnessing the capabilities of UV–Visible spectroscopy, researchers can gain valuable insights into the electronic properties, concentration, and purity of samples, advancing their understanding and applications in fields such as chemistry, biochemistry, and material science.

### 3.5 Nuclear Magnetic Resonance Spectroscopy (NMR)

Nuclear magnetic resonance spectroscopy (NMR) is a powerful analytical technique widely utilized in chemistry, biochemistry, and related fields for the structural elucidation and characterization of organic compounds, as well as the investigation of molecular dynamics and interactions (Bruno et al. 2023).

NMR exploits the inherent magnetic properties of atomic nuclei to provide valuable information about the structure and behavior of molecules. It involves subjecting a sample to a strong magnetic field and applying radiofrequency pulses to induce nuclear spin transitions. By measuring the absorption and emission of energy during these transitions, detailed information about the chemical environment, connectivity, and dynamics of the sample can be obtained (Tampieri et al. 2021).

One of the primary advantages of NMR is its non‐destructive nature. It requires minimal sample preparation, allowing for the analysis of various sample types, including liquids, solids, and gases, without altering their chemical composition. This makes NMR an invaluable tool in the characterization of complex mixtures and natural products (Wishart et al. 2022).

NMR provides high‐resolution structural information, enabling the determination of molecular connectivity and spatial arrangement of atoms within a molecule. It can identify functional groups, differentiate between isomers, and elucidate complex molecular architectures. Additionally, NMR techniques such as two‐dimensional (2D) NMR and multi‐dimensional NMR experiments offer enhanced spectral resolution and provide detailed insights into molecular structure and dynamics (Sahoo et al. 2020).

Furthermore, NMR spectroscopy is capable of quantifying the abundance of different molecular species in a mixture. By integrating the area under specific peaks in the NMR spectrum, the relative concentrations of compounds can be determined, facilitating quantitative analysis and metabolic profiling (Giraudeau 2023).

NMR spectroscopy also enables the study of molecular interactions and dynamics (Alderson and Kay 2021). Through techniques such as diffusion NMR (Thomlinson et al. 2022), relaxation NMR (dos Reis Lino et al. 2021), and nuclear Overhauser effect spectroscopy (NOESY) (Madhu 2023), researchers can investigate the movement, rotation, and interaction of molecules, as well as the binding affinity between ligands and receptors. This information is valuable in fields such as drug discovery, where understanding molecular interactions is crucial.

In recent years, advances in NMR technology, including high‐field magnets (Yanagisawa et al. 2022) and cryogenic probes (Matsuki et al. 2022), have significantly enhanced the sensitivity and resolution of NMR spectra. This allows for the analysis of smaller sample volumes and detection of low‐concentration compounds, broadening the scope of NMR applications.

NMR spectroscopy is a highly effective analytical technique used for structural elucidation, quantitative analysis, and studying molecular interactions and dynamics. Its non‐destructive nature, high resolution, and versatility make it an essential tool across various scientific disciplines. NMR spectroscopy enables researchers to gain deep insights into the structure, composition, and behavior of molecules, thus advancing knowledge and applications in chemistry, biochemistry, and related fields.

## 4 Identification Techniques of Bioactive Compounds

### 4.1 High‐Resolution Mass Spectrometry (HRMS)

High‐resolution mass spectrometry (HRMS) is an advanced analytical technique that has revolutionized the field of compound identification and characterization (Razgonova et al. 2023). It combines the use of high‐resolution mass analyzers with sophisticated data acquisition and processing techniques, enabling precise determination of molecular weights and accurate mass spectra. HRMS has become an indispensable tool in various scientific disciplines, including chemistry, biochemistry, pharmaceuticals, and environmental analysis (Alshammari et al. 2023).

One of the key advantages of HRMS is its ability to provide highly accurate mass measurements (Nehmeh et al. 2023). Traditional mass spectrometry techniques have limited resolution, making it challenging to differentiate compounds with similar masses. HRMS overcomes this limitation by offering significantly higher resolution, allowing for clear distinction and accurate determination of molecular weights (Lai and Wang 2023). This capability is particularly valuable in the identification of unknown compounds, as it provides crucial information for structural elucidation and database searching.

HRMS can be combined with various ionization techniques, such as electrospray ionization (ESI) or matrix‐assisted laser desorption/ionization (MALDI), to ionize samples for analysis (Letourneau and Volmer 2023). The choice of ionization technique depends on the nature of the sample and the desired analyte class. The ability to ionize a wide range of compounds makes HRMS applicable to diverse sample types, including small organic molecules, peptides, proteins, lipids, and metabolites (Deschamps et al. 2023).

In addition to accurate mass measurement, HRMS also provides detailed mass spectral information. The high resolution and mass accuracy enable the detection of isotopic patterns, fragmentation patterns, and elemental compositions of ions (Jongedijk et al. 2023). This information can be used to elucidate the structure and connectivity of compounds (Kajtazi et al. 2023), identify functional groups (Latz et al. 2023), and differentiate between isomeric species (Akhlaqi et al. 2023). Furthermore, HRMS can be combined with tandem mass spectrometry (MS/MS) techniques to provide additional structural information and enhance compound identification capabilities.

Recent advancements in HRMS technology have significantly improved instrument sensitivity, resolution, and speed. High‐field magnets, improved ion sources, and innovative mass analyzers have enhanced the overall performance of HRMS instruments (Deschamps et al. 2023). These advancements have allowed for the analysis of smaller sample volumes, detection of low‐abundance compounds, and improved throughput in data acquisition (Song, Baral, et al. 2023). Moreover, advancements in data processing and computational approaches, including machine learning and artificial intelligence, have further enhanced the speed and accuracy of compound identification using HRMS data.

Therefore, High‐Resolution Mass Spectrometry (HRMS) is a powerful and versatile analytical technique that has transformed compound identification and characterization. Its ability to provide highly accurate mass measurements, detailed mass spectral information, and the integration of advanced data analysis tools has made HRMS an essential tool in various scientific fields. By harnessing the capabilities of HRMS, researchers can efficiently identify and characterize unknown compounds, elucidate their structures, and advance knowledge and applications in chemistry, biochemistry, pharmaceuticals, and environmental analysis.

### 4.2 Chemoinformatics and Machine Learning

Chemoinformatics and machine learning have emerged as powerful tools in the identification and characterization of bioactive compounds. By combining computational methods with chemical information, these approaches offer efficient and accurate means of predicting and analyzing the properties and activities of compounds, aiding in drug discovery, toxicity assessment, and molecular design (Bajorath et al. 2022).

Chemoinformatics involves the application of statistical and computational techniques to chemical data, enabling the analysis, interpretation, and prediction of chemical properties and behaviors. By leveraging large databases of chemical structures and associated properties, chemoinformatics methods can identify patterns, relationships, and trends in chemical data. This information is crucial for understanding structure–activity relationships (SAR) and predicting the bioactivity of compounds (Niazi and Mariam 2023).

Machine learning algorithms play a vital role in chemoinformatics by learning from data and making predictions or decisions without explicit programming. These algorithms are trained using chemical descriptors, molecular fingerprints, or other chemical representations as input features. By utilizing large and diverse datasets, machine learning models can extract meaningful information and patterns, enabling the prediction of various properties and activities of bioactive compounds (Park et al. 2022).

One of the primary applications of chemoinformatics and machine learning is virtual screening, which involves the rapid identification of potential bioactive compounds from large compound libraries (Bhowmik et al. 2023). By training machine learning models on known bioactive compounds, these models can predict the bioactivity of new compounds and prioritize those with high potential for further experimental investigation. This approach accelerates the drug discovery process by focusing on the most promising candidates and reducing the number of compounds requiring experimental screening (Medina‐Franco 2021).

Chemoinformatics and machine learning methods are also widely used for toxicity prediction and assessment. By analyzing chemical structures and incorporating toxicological data, these approaches can predict the toxicity profiles of compounds, aiding in the selection of safer and more effective candidates for drug development or other applications. This is particularly valuable in the early stages of compound screening, where identifying potential toxic compounds can save time, resources, and prevent harmful effects (Raslan et al. 2023).

To support cheminformatics and machine learning analyses, large chemical databases, molecular libraries, and online resources are available. These resources provide access to chemical structures, biological activity data, and other relevant information, allowing researchers to extract knowledge and build robust models for compound identification and prediction (Martinez‐Mayorga et al. 2020).

Finally, chemoinformatics and machine learning have significantly contributed to the identification and characterization of bioactive compounds. By integrating computational methods, chemical information, and large datasets, these approaches enable the prediction of bioactivity, toxicity, and molecular properties of compounds. The applications of chemoinformatics and machine learning extend to virtual screening, toxicity assessment, and compound design, accelerating drug discovery and facilitating the development of bioactive compounds with therapeutic potential.

### 4.3 Multi‐Dimensional Analysis

Multi‐dimensional analysis has emerged as a powerful approach in the study of bioactive compounds, enabling researchers to gain a more comprehensive understanding of their properties, interactions, and biological activities (Yang et al. 2023). By combining multiple analytical techniques and data sets, multi‐dimensional analysis offers enhanced resolution, sensitivity, and information extraction capabilities (Wang, Yang, et al. 2023).

One key aspect of multi‐dimensional analysis is the integration of complementary analytical techniques. By combining techniques such as mass spectrometry (MS), NMR spectroscopy, and chromatography, researchers can obtain a wealth of structural and functional information about bioactive compounds (Meheuddin and Kazerooni 2024). Each technique provides unique insights into different aspects of the compound, and their integration allows for a more holistic characterization.

For example, multi‐dimensional liquid chromatography (LCxLC) combines two or more chromatographic separation modes, such as reversed‐phase and normal‐phase chromatography, to achieve higher resolution and increased peak capacity (Pardon et al. 2024). This enables the separation of complex mixtures and the identification of closely related compounds that may have been challenging to resolve using a single chromatographic method. LCxLC coupled with MS or NMR detection provides not only separation information but also structural insights and identification capabilities, facilitating the characterization of bioactive compounds (Wysor 2023).

Multi‐dimensional analysis also extends to the use of advanced data acquisition and processing techniques. For instance, two‐dimensional nuclear magnetic resonance spectroscopy (2D NMR) allows for the correlation of signals from different nuclei in a molecule, providing valuable structural information about connectivity and spatial arrangements (Lapina and Yakovlev 2023). By combining 2D NMR with other techniques like MS or LC, researchers can obtain a more comprehensive picture of the compound's structure and properties (Duarte et al. 2023). By utilizing advanced algorithms and data mining techniques, researchers can extract meaningful information from multi‐dimensional data sets and unravel complex relationships between the structure, properties, and activities of bioactive compounds.

Overall, multi‐dimensional analysis represents a paradigm shift in the characterization and understanding of bioactive compounds. By integrating complementary techniques, advanced data acquisition and processing, and computational approaches, researchers can obtain a more comprehensive view of the compounds' properties, interactions, and biological activities. This approach holds significant potential for advancing drug discovery, chemical biology, and the development of bioactive compounds with therapeutic applications.

## 5 Food Bioactive Components Delivered Using Stimuli‐Responsive Carrier

**FIGURE 4:**
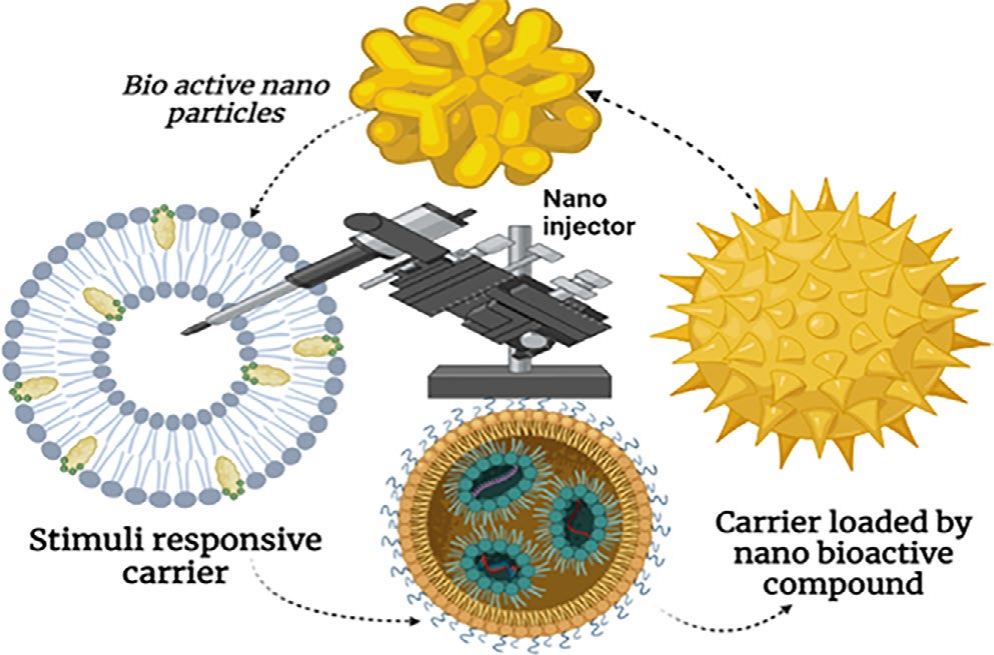
Stimuli‐responsive carrier for nano bioactive component.

Recent advancements in nanotechnology and materials science have spurred the development of various nanocarriers aimed at efficiently delivering bioactive phytochemicals. Among the most studied carriers are liposomes, emulsions, micelles, hydrogels, coacervates, polymeric nanoparticles, and conjugates. These delivery systems offer significant potential for enhancing the efficacy of nutraceuticals due to their ability to improve the physical properties of encapsulated bioactive compounds, such as solubility and stability. They also protect sensitive phytochemicals from harsh gastrointestinal conditions, extend systemic circulation, prevent the degradation of fragile compounds, and promote cellular uptake. Additionally, these systems facilitate targeted delivery to specific tissues or organs. Recent developments have focused on stimuli‐responsive carriers, which are designed to provide precise control over the timing, location, and dosage of release, thereby minimizing side effects. These advanced systems, influenced by progress in food science, functional foods, nutraceuticals, and materials science, enable on‐demand release of bioactive compounds triggered by physical, chemical, or biological stimuli from the microenvironment. The release mechanisms include hydrolysis, protonation, redox reactions, isomerization, and carrier disassembly, triggered by endogenous stimuli such as pH, enzymes, redox potential, and temperature, or exogenous factors like light, magnetic fields, ultrasound, and irradiation (Alsehli 2020; Gu et al. 2018; Muntimadugu et al. 2015). Stimuli‐responsive carriers may be single‐stimulus, reacting to one type of stimulus, or multi‐stimuli‐responsive, activated by multiple stimuli (Chen et al. 2020; Patra et al. 2018; Rao et al. 2018). Often referred to as “smart” delivery systems, these carriers combine the properties of nanohydrogels, micelles, graphene nanoparticles, solid nanoparticles, nanoliposomes, and polymeric nanoparticles with the ability to respond dynamically to stimuli such as pH, temperature, redox conditions, or enzymes. They are engineered for precise site‐specific targeting, including targeting specific organs or tumor microenvironments. The effectiveness of these systems hinges on the chemical bonds, functional groups, or polymers used to facilitate the stimuli‐responsive function and trigger release (Kang et al. 2020). Consequently, these advanced carriers hold great promise for reducing toxic side effects, enabling controlled release, enhancing site‐specific targeting, and improving cellular uptake (Figure 4).

## 6 Emerging Applications of Bioactive Compounds

Biologically active compounds are secondary metabolites that are extracted from plant, animal, fungal, or microbial sources. In addition to their medicinal and toxic effects, these compounds possess new properties that led to the expansion of their applications in several fields (cosmetics, biofuels, and functional compounds). Currently, these compounds are extracted using modern methods such as (supercritical fluid extraction, microwave extraction, pulsed electric field extraction, enzyme extraction, ultrasonic extraction, or pressure extraction), which have enhanced their stability while preserving their biological and functional diversity (Pai et al. 2022). Phenolic compounds are widely used as alternative therapeutic agents, and polyphenolic compounds from natural sources have been used in many fields in order to develop new products or technologies and reduce environmental risks resulting from them and their negative impact on human health (Dias et al. 2021), (Tables 1 and 2). Latest studies have proven that the mechanism of protective consequences of polyphenols happens through mobile signaling pathways and is not always immediately brought on by epigenetic movements within the context of pathological physiological constraints. In food technology, they are being used to enhance the nutritional profile of functional foods, contributing to improved health outcomes by providing antioxidant, anti‐inflammatory, and antimicrobial properties. These compounds are also increasingly incorporated into active packaging materials, extending the shelf life of perishable goods by preventing microbial growth. Recent research has proven that (Corrêa et al. 2018; Daglia 2020). The textile industry is one of the most polluting, generating many chemicals, like synthetic dyes, and quite several colors that range from yellow to pink, and for that reason, they have been exploited in the development of natural dyes with less toxicity than the traditional ones used in silk and wool fabric dyeing (Dias et al. 2021). In the field of pharmaceuticals, bioactive compounds such as polyphenols, flavonoids, and alkaloids are being investigated for their potential in developing new drugs for chronic diseases, including cancer, diabetes, and cardiovascular conditions. Their ability to target specific biological pathways with minimal side effects has made them a promising alternative to synthetic drugs. Additionally, cosmetic industries are leveraging bioactive compounds for their anti‐aging, moisturizing, and skin‐protective properties, creating products that support skin health and reduce the signs of aging. The recent surge in interest also includes their use in nanotechnology for drug delivery systems, enhancing the bioavailability and targeted release of therapeutic agents. As research continues, these natural compounds are proving to be vital in promoting health and sustainability across various sectors. Tables 3, 4 and 5 represent food applications of bioactive compounds, medical applications of bioactive compounds, and industrial and biological applications of bioactive compounds, respectively.

**TABLE 2:** The pH‐responsive carriers and polymeric nanoparticle‐based carriers.

Carriers	Types	References
1‐Acid‐labile chemical bonds 2‐Ionizable chemical groups 3‐pH‐sensitive polymers 4‐pH‐sensitive peptides 5‐Gas‐generating precursors 6‐ Hydrogels	[e.g., acetal, hydra‐zone, ortho‐ester, imine, and amide bonds] [e.g., amines, carboxylic acids, and phosphoric acids] [e.g., chitosan, gelatin, and cyclodextrin] (e.g., histidine, chitosan, alginate, carrageenan) (e.g., sodium bicarbonate, ammonium‐bicarbonate, and alkoside) (e.g., Tremella polysaccharides, carboxymethyl cellulose, and nonionic surfactants as the main hydrogel building blocks, carboxymethyl chitin)	(Shishir et al. 2021; Do et al. 2022; Kropacheva et al. 2023) (Zhang et al. 2021; Rezaei et al. 2022; Donders et al. 2023) (Xiong et al. 2020; Gutierrez et al. 2022; Shen et al. 2023) (Du et al. 2022; Suo et al. 2021; Rigogliuso et al. 2023) (Liu et al. 2022; Singh et al. 2021; Zhao et al. 2023; Yu et al. 2023) (Zhao et al. 2023; Liao and Huang 2022; Hilal et al. 2023)
**Nano carriers**	**Encapsulated materials**	**References**
1‐ Okra polysaccharides with gelatin 2‐ Sodium carboxymethyl cellulose 3‐ Chitosan 4‐ Nano emulsions (Gums) 5‐ Alginate	Encapsulation of iso‐quercetin Encapsulation of Lactic acid Encapsulation of oleuropein from olive Citral, β‐carotene, tributyrin, flaxseed oil, coenzyme Q capsaicin, and several oil‐soluble vitamins Vitamins, minerals, essential fatty acids, peptides, essential oils, bioactive oils, polyphenols and carotenoids	(Smola et al. 2008; Li et al. 2020a) (Gunathilake et al. 2020) (Lu et al. 2021) (Singh et al. 2023) (Karim et al. 2022)

*Note:* pH‐responsive carriers, they can trigger gate release of the encapsulated bioactive compound at the target site; Polymeric nanoparticle‐based carriers; The decrease in particle size leads to an increase in surface area, leading to an enhanced dissolution rate. Polymeric nanoparticles that are stable in acidic conditions have also been developed.

**TABLE 3:** Food applications of bioactive compounds.

Mechanism	Food applications	Phenolic compound		References
Added value to human health protection	Natural food additives, nutritional supplements, producing functional products, food coloring, meat and meat products	Bioactive compounds		Dias et al. (2021) Caleja et al. (2017) Talekar et al. (2018) Chhikara et al. (2019) Pogorzelska‐Nowicka et al. (2018)
An alternative to synthetic additives (0.5%)	Additive materials	Natural extracts	Natural extracts	Câmara et al. (2020)
Prevention lipid oxidation just like (BHT, 100 Ppm)	Sheep meat nuggets preservatives	Lychee pericarp extract (1.5%)		Das et al. (2016)
Rich in phenolic acids, and the effect of it is similar to the artificial additives	Mayonnaise preservation	Rice ethanolic extract (2%)		Câmara et al. (2020)
Inhibited *Staphylococcus aureus* , *Coliforms*, and mesophyll bacteria and also the growth of molds and yeasts	Antibacterial	Natural Additive		Martillanes et al. (2020)
Stable red‐purple Color, Increased antioxidant activity and decreased microbiological degradation	Natural colorants (E163), fresh sausage preservation, indicators of fish freshness, and anti‐lipidic peroxidation agents in olive oil packing	Anthocyanins		Albuquerque et al. (2021) Baldin et al. (2016) Zeng et al. (2019) Wang, Xia, et al. (2019) Wang, Guo, et al. (2019)
Antioxidant activity and antimicrobial Activity against *Escherichia coli* and *Listeria innocua*	Edible films	Tannin‐Protein		Cano et al. (2020)
Food enhancer	Food additives	Carotenoids Curcumin		
Flavoring agents	Food additives	Vanillin and Cinnamaldehyde		
An alternative source for protein	Food supplement	Single‐cell proteins		Ritala et al. (2017)
Baking and in the beverage Industry	A stabilizer	Laccase		Mayolo‐Deloisa et al. (2020)
Coloring agent	Food coloring	Astaxanthin		Gwaltney‐Brant (2021)
Emulsifier, thickener, and stabilizer	Food additives	Xanthan		Habibi and Khosravi‐Darani (2017)
Prevent oxidation in foods	An additive	Ascorbic acid	Acids	Pai et al. (2022)
Acidulation and preservation	Food additives	Lactic acid		Miller et al. (2011)
Food preservative and flavoring agent	Food additives	Citric acid		Pai et al. (2022)
An acidulant	Food additives	Fumaric acid		Pai et al. (2022)

**TABLE 4:** Medical applications of bioactive compounds.

Mechanism or effective materials	Medical applications	Bioactive compounds		References
Biological potentialities, skin protection, anti‐aging activities, absorb UV radiation, and prevent solar radiation from penetrating the skin	Anti‐aging creams and sunscreens (increased skin elasticity, reducing pores and skin roughness and wrinkles after 60 days of use), hair, nail care products, and cosmetics. Reduced progression onset and persistent sicknesses related to oxidative stress, cardiovascular and age‐associated diseases, kind 2 diabetes, and cancer, when Polyphenols consumption at 1 g/day	Polyphenols	Bioactive compounds	Dias et al. (2021) Câmara et al. (2020)
Biological activity (antimicrobial, antioxidant, and anti‐inflammatory activity), bioactive compounds necessary for the human body as mentioned before. Hydrocarbon degradation	Positive results on health. Form of coagulants. Form of biofilms Form of bioactive Extracts. Wastewater treatment	Bioactive compounds, Phytochemicals, (polyphenols, alkaloids, sulfur‐conserving compounds, and terpenoids)		Câmara et al. (2020) Pai et al. (2022) Ibrahim et al. (2021) Mugge et al. (2021) Zerrifi et al. (2018)
Antioxidant and anti‐tyrosine and anti‐inflammatory activities	Stable bioactivities cosmetic cream	Phenolic Acids, P‐Hydroxybenzoic, P‐Coumaric, and Protocatechuic Acids		Kamma et al. (2016) Taofiq et al. (2019)
Remove turbidity and flocculate suspended solids	Wastewater treatment Plants	Tannins		Ibrahim et al. (2021)
Remove anionic dyes and cationic from water	Water treatment	Tannins and Tannic acid of *Acacia mearnsii*		Grenda et al. (2020)
Remove the heavy metals and methylene blue	Treatment of contaminated water	Tannin cryogels and Wattle tannins		Das et al. (2020)
Consisting of pseudoprototinosaponin‐ AIII and prototinosaponin‐AIII, alkaloids, triterpenes, thiocyanates, and cardiac glycosides	Antidiabetic properties	*Aloe vera* extract	Natural plant extracts	Shrinet et al. (2021)
Cyanogenic glycosides	Antidiabetic properties	*Terminalia catappa* extract		Behl and Kotwani (2017)
Steviol glycosides	Antidiabetic properties	*Stevia rebaudiana* Leaves extract		Žlabur et al. (2015)
A flavone, Baicalein	Anticancer and anti‐high‐field inflammatory Activities and has been used to treat several Gastrointestinal ailments such as gastric ulceration, Liver fibrosis	Extract of Dried roots of *S. baicalensis Georgi*		Xie et al. (2019)
Silymarin	Treatment of Liver disorders as well as antitumor activity	*Silybum marianum* L. *Gaertner* extract		Wianowska and Wiśniewski (2015)
Nutraceuticals (quercetin and kaempferol)	Skin hyperpigmentation Conditions and alzheimer, and neurodegenerative disorders	*Anthemis cotula L*. extract (stinking *chamomile*)		Sut et al. (2019) Makkar et al. (2020)
Important sources of: Malic acid 2‐Hydroxy‐3‐(2‐hydroxyphenyl) Propanoic acid Trihydroxy‐octadecadienoic acid Caffeic acid Quinic acid, 5‐p Coumaroylquinic acid Chlorogenic acid 5‐Feruloylquinic acid Catechin Hexose Feruloylglycoside Kaempferol‐xylose Kaempferol‐ Rhamnoside Naringenin Hexoside Kaempferol‐glucoside Quercetin‐3‐O‐glucoside Taxifolin hexoside Caffeoyl derivative hexose Kaempferol‐hexose malic acid Procyanidins B2 Kaempferol‐3‐Orutinoside Rutin Ursolic acid/Oleanolic acid	Therapeutic effects	*Eriobotrya japonica Lindl*. Leaves extracts		Silva et al. (2020)
Rich in α‐linolenic, oleic, and palmitic acids from *Botryococcus braunii* , diethyl phthalate	Activity against harmful algal blooms (HAB)	*Stoechospermum marginatum*		Zerrifi et al. (2018, 2021)
Important sources of bioactive compounds, sush as (Fucose‐sulfated polysaccharides extracted from brown algae ( *A. nodosum* and *Fucus vesiculosus* ))	Antiviral, anti‐inflammatory, antioxidants and anticoagulants	Algae and marine organisms	Algae and marine organisms	Rodriguez‐Jasso et al. (2011) Garcia‐Vaquero et al. (2020)
Fucoidans (fucose, uronic acids, galacturonic acid, glucuronic acid, sulfates)	Antiviral, anti‐inflammatory, antioxidants and anticoagulants	Brown algae		Sichert et al. (2021)
Rich in Phlorotannins	Treatment of rheumatoid arthritis, goiter, asthma, and obesity	*Fucus vesiculosus*		Catarino et al. (2019)
Activity against the *Herpes simplex* virus (HSV)	Therapeutic effects	*Chondrus crispus* and *Codium fragile* extracts		Kulshreshtha et al. (2015)
Important sources of: Tangeretin 5′‐demethyltangeretin Nobiletin 3′‐demethylnobiletin 4′‐demethylnobiletin 3′4′‐demethylnobiletin 5‐demethylnobiletin 5,3′‐demethylnobiletin 5,4′‐demethylnobiletin 5,3′,4′‐demethylnobiletin Naringenin Hesperetin	Therapeutic effects	Citrus	Fruits	Li et al. (2020b)
Sources of antibiotics (penicillins, carbapenems, and cephalosporins), and Compactin and lovastatin	Antibiotics, cholesterol‐lowering agents, antibacterial, antiviral, anti‐cancer, and immunostimulants	Fungi	Microorganisms sources	Hoeksma et al. (2019) Poojary et al. (2017)
Sources of monocerin	A biopesticide. Bioremediation of methyl tert‐butyl ether. A soil contaminant. a gasoline additive	Fungi, Drechslera (strain 678)		d'Errico et al. (2021)
Important sources of alkaloid compounds	Antibiotic potential	Bacteria ( *Pseudomonas aeruginosa* UWI‐1)		Ramkissoon et al. (2020)
Lipopeptides	Recovery of motor oil from contaminated soil and degradation of polycyclic aromatic hydrocarbons	*Bacillus subtilis CN2*		Bezza and Chirwa (2015)
Scleritodermin A	Human colon, breast, and ovarian tumors treatment	*Scleritoderma nodosum*		Sinha and Hader (2021)
Sources of high‐value antioxidants	Therapeutic effects	Remnants of bass and bream sea (gills, head, bones)	Animal sources	Franco et al. (2020)
Sources of beneficial unsaturated fatty acids	Prevention of cardiovascular and cerebrovascular diseases	*Rana chensinensis* ovum oil		Gan et al. (2020)
Development of antivenom	Therapeutic effects	Venom of snake		Abd El‐Aziz, Jaquillard, et al. (2020), Abd El‐Aziz, Soares, and Stockand (2020)

**TABLE 5:** Industrial and biological applications of bioactive compounds.

Mechanism	Industrial and biological applications	Phenolic compound		References
The ability of it to form complexes of proteins and metal ions	Water treatment contaminated with heavy metals, surfactants, pharmaceutical compounds, and dyes	Tannins		Dias et al. (2021) Bacelo et al. (2016)
Biological potentialities, skin protection, anti‐aging activities, absorb UV radiation, and prevent solar radiation from penetrating the skin	Water treatments and natural dyes for textiles	Polyphenols		Dias et al. (2021)
Hydrocarbon degradation	Wastewater treatment Polymers, biomaterials, textiles dyes, leather, cosmetics, perfumes, oil and soap industries	Bioactive compounds	Bioactive compounds	Pai et al. (2022) Nogueira et al. (2020) Agnhage et al. (2017) Das et al. (2020) Sharmeen et al. (2021) Ng et al. (2021)
Removal turbidity and flocculate suspended solids	Wastewater treatment Plants	Tannins		Ibrahim et al. (2021)
Removal anionic dyes and cationic from water	Water treatment	Tannins and Tannic acid of *Acacia mearnsii*		Grenda et al. (2020)
Removal the heavy metals and methylene blue	Contaminated water Treatment	Tannin cryogels and Wattle tannins		Das et al. (2020)
—	UV light protection in textiles, flame retardancy, and antibacterial	Tannin‐based macromolecules		Basak et al. (2021)
—	Uv protection by dyeing cotton by phenolic tea extracts	Tea phenolic extracts	Natural extract	Bonet‐Aracil et al. (2016)
Sources of tannins	Natural dyes	Acacia extract, oak galls extract, and quebracho extract		Câmara et al. (2020)
Anthocyanins	Natural dyes	Sorghum extract		Câmara et al. (2020)
Rich in tannins and flavonoids	Improve the sun protection factor of a cream	A *nephelium lappaceum* peel extract		Mota et al. (2020)
Rich in epigallocatechin‐3‐gallate, due to its effects on dermal papilla cells	Hair care niche	Green tea		Kwon et al. (2007)
Hair increase‐promoting pastime after chemotherapy‐brought on alopecia	Hair therapeutic benefits	Procyanidinic extract of *Malus Pumila* Miller cv Annurca		Riccio et al. (2018)
Sources of monocerin	A biopesticide. Bioremediation of methyl tert‐butyl ether. A soil contaminant. a gasoline additive	Fungi, Drechslera (strain 678)	Microorganisms sources	d'Errico et al. (2021)
Lipopeptides	Recovery of motor oil from contaminated soil and degradation of polycyclic aromatic hydrocarbons	*Bacillus subtilis CN2*		Bezza and Chirwa (2015)
Run dye	Colorants	*Miscanthus sinensis* Andersson		Pinzon et al. (2020)
Anthraquinone dyes	Colorants	*Rubia tinctorum L*.		Agnhage et al. (2017)
Sources of lipids, microbial oils and single‐cell oils	Production alternative fuels	Yeasts (sush as oleaginous yeast), fungi, microalgae, and bacteria		Gorte et al. (2020)
Sources of (camphor, eucalyptol, limonene, β‐pinene)	Production renewable energy	*C. camphora*		Zhang, Wu, Gao, et al. (2020), Zhang, Wu, Lai, et al. (2020)
Enhanced power and improves electricity production	Generate electricity	Microbes, and Microbial fuel cells		Nath and Ghangrekar (2020)
Sources of sugar, Starch, cellulosic, fiber, and glycerine	Production biofuels bioenergy and (such as ethanol, butanol and methanol)	Plant waste, animal waste, and food industry waste	Waste	Swain (2017)
Condensation processes of β‐pinene	Production diesel and renewable fuel	β‐pinene		Jung et al. (2016)
Regulating the release of antioxidants (tannin and lipoic acid)	Biomedical and cosmetic Applications	A biomaterial (collagen, polyurethane, and cellulose)		Spiridon et al. (2020)
Encapsulation of antioxidants with polyethylene oxide nanofibers	Encapsulation of antioxidants	*Aloe vera* agrowastes		Solaberrieta et al. (2020)

## 7 Conclusions

In conclusion, the extraction, isolation, identification, and characterization of bioactive compounds from plant‐based foods are crucial for leveraging their potential across various applications. Advanced extraction techniques, such as UAE, supercritical fluid extraction, and MAE, have markedly improved both the efficiency and yield of these compounds. Methods for isolation and purification, including chromatography, crystallization, and membrane filtration, enable the acquisition of pure, high‐potency bioactive compounds. Characterization techniques like spectroscopy (UV–Vis, FTIR, NMR), mass spectrometry, and X‐ray diffraction offer essential insights into the structural properties and functional groups of these compounds. The bioactive compounds identified often possess a range of beneficial characteristics, including antioxidant, antimicrobial, anti‐inflammatory, anticancer, and neuroprotective properties, positioning them as valuable candidates for applications in pharmaceuticals, nutraceuticals, cosmetics, and the food industry. Emerging uses include natural preservatives in food products, functional ingredients in dietary supplements, and active components in skincare formulations. Additionally, advancements in novel delivery systems, such as nanoparticles and encapsulation technologies, have enhanced the bioavailability and stability of these compounds, broadening their potential applications. Ongoing research and technological progress in these areas promise to address health, environmental, and economic challenges across various industries.

## Author Contributions

**Tarek Gamal Abedelmaksoud:** conceptualization; investigation; methodology; writing – original draft; formal analysis; supervision; resources; data curation. **Mohamed Ibrahim Younis:** writing – review editing; validation; visualization. **Ammar B. Altemimi:** conceptualization; writing – review editing; validation; visualization. **Rawaa H. Tlay:** conceptualization; writing – review editing; validation; visualization; methodology. **Nora Ali Hassan:** writing – review editing; validation; visualization.

## Conflicts of Interest

The authors declare no conflicts of interest.

## Data Availability

Data sharing is not applicable to this study, as no new datasets were generated or analyzed during the research.
